# *Klebsiella pneumoniae* Renal Abscess Syndrome: A Rare Case with Metastatic Involvement of Lungs, Eye, and Brain

**DOI:** 10.1155/2013/685346

**Published:** 2013-08-05

**Authors:** Divyanshu Dubey, Fayez S. Raza, Anshudha Sawhney, Ambarish Pandey

**Affiliations:** ^1^Department of Internal Medicine, University of Texas Southwestern Medical Center, 5323 Harry Hines Boulevard, Dallas, TX 75235, USA; ^2^Netaji Subhash Chandra Bose Medical College, Jabalpur, India

## Abstract

We describe a rare case of *Klebsiella pneumoniae* renal abscess with metastatic spread leading to endopthalmitis, pulmonary cavitary lesions, and cerebral emboli in a 41-year-old Hispanic female with diabetes mellitus who presented with a four-to-five-day history of fevers, headache, eye pain, and vomiting. She was treated with IV antibiotics and made a gradual but full recovery.

## 1. Introduction

*Klebsiella pneumoniae*, a member of the Enterobacteriaceae family, is a virulent Gram negative organism that causes nosocomial infections. It has a higher tendency to infect immunocompromised patients including those with diabetes. Commonly attributed infections to *Klebsiella pneumoniae* include urinary tract infections (UTIs) and pneumonias. Rarely, incidence of abscess formation secondary to *Klebsiella pneumonia* infection has been reported in organ like liver, lung, and brain [1]. In addition, certain serotypes of *Klebsiella pneumoniae*, particularly K1 and K2, have been reported to involve secondary areas of the body through metastatic spread from the primary abscess [1–8]. *Klebsiella* liver abscess presenting as a widely metastatic invasive syndrome has been reported in South East Asia [1, 2]. However, only a few cases of renal abscess with metastatic spread have been reported [2–8].

In this report, we describe a rare case of *Klebsiella pneumoniae* renal abscess with metastatic lesions to the brain, eyes, and lungs.

## 2. Case Report

**Figure 1:**
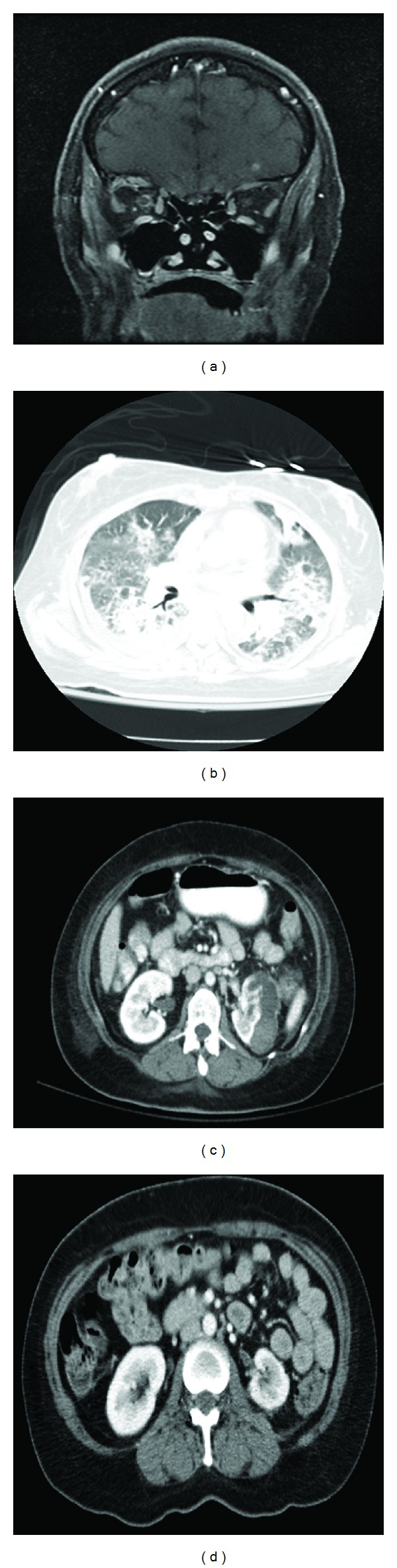
(a) Multifocal contrast enhancing lesions consistent with septic embolic, inflammatory changes surrounding the right ocular globe and enhancement surrounding the right optic nerve. (b) CT chest with IV contrast showing bilateral parenchymal consolidative changes with cavitary lesions. (c) CT abdomen/pelvis with IV contrast depicting left renal abscess, with the largest component in a subcapsular location. (d) Minimal amount of residual fluid adjacent to the left kidney after drainage and systemic antibiotic therapy.

A 41-year-old Hispanic female with a history of type 2 diabetes mellitus presented with four-to-five days of progressively worsening fever, headache, right eye pain, blurred vision, nausea, and vomiting. She also complained of shortness of breath and pleuritic chest pain over the last twenty four hours. On examination, a hypopyon was visualized in the anterior chamber of right eye, and bilateral crackles were heard on lung auscultation. No significant weakness or numbness was found on neurological exam. Complete blood count showed leukocytosis with neutrophilic predominance. Urine analysis was consistent with urinary tract infection. Her condition deteriorated at this point, and she became confused and disoriented. Cultures were obtained, and she was started on empiric intravenous antibiotics (Vancomycin and Zosyn). Ophthalmology service was consulted, and she was treated with intravitreal injection of vancomycin and ceftazidime. MRIs of her brain and orbits were obtained which showed inflammatory changes surrounding the right ocular globe, consistent with endopthalmitis (Figure 1(a)). There were also multifocal regions of increased FLAIR signal within the cortex consistent with septic embolic disease. CT of her chest revealed multiple cavitary lesions, likely secondary to embolic phenomenon (Figure 1(b)). Given the embolic involvement of lung, brain, and eyes, a workup to assess the possible source of infection was conducted. The patient underwent trans-thoracic and transesophageal echocardiographies which were both negative for endocarditis. She had no signs or symptoms consistent with thrombophlebitis (Lemierre's syndrome) as a source of embolic disease.

On the third day of admission, patient's urine culture grew *Klebsiella pneumoniae*. CT scan of the abdomen showed a left renal abscess, with the largest component in subcapsular location (Figure 1(c)). The patient underwent CT guided drainage of the renal abscess and culture of the collected specimen grew *Klebsiella pneumoniae* (serotype K1) which was resistant to most antibiotics except the carbapenems. She was started on intravenous meropenem (2 grams every 8 hours) for a duration of 8 weeks. After a 10-week complicated hospital course, which included ICU care, the patient recovered and was able to be discharged home. Follow-up imaging of her abdomen showed resolution of her previously seen renal abscess (Figure 1(d)).

## 3. Discussion

Our case emphasizes the importance of considering *Klebsiella pneumoniae* abscess as a part of differential diagnosis in patients presenting with multiorgan pathology concerning septic/embolic phenomenon. While *Klebsiella* liver abscess with septic emboli has been well reported, only few cases of *Klebsiella pneumoniae* renal abscess with septic emboli have been published.

A case series by Chang et al. described 24 cases of *Klebsiella* renal abscess in Taiwan. Of the twenty four cases, only three of these patients were mentioned to have metastatic lesions from a renal abscess [3]. We found 4 case reports of endophthalmitis secondary to *Klebsiella pneumoniae* infection. A single case was reported by Deryckère et al., while Chen et al. reported two cases of septic involvement of the eyes [4, 5]. A case of endopthalmitis along with ecthyma gangrenosum was published by Stokta and Rupp [6]. Finally, a case of *Klebsiella* renal abscess complicated by endopthalmitis, diabetic ketoacidosis, and disseminated intravascular coagulation was reported from Japan [7].

Several potential risk factors for the development of perinephric and renal abscesses have been postulated. These include urolithiasis, recurrent urinary tract infections, history of urologic surgery, structural abnormalities of the urinary tract, trauma, and diabetes [2, 8]. In addition, capsular serotypes K1 and K2 are the most common forms seen in *K. pneumoniae* abscess, as reported in a seroepidemiologic study [1]. Serotype K1, as in our patient, has been associated with liver abscess and subsequent development of endopthalmitis and meningitis, especially in diabetic patients [1, 2].

As the cases are now being reported from outside South East Asia, *Klebsiella pneumoniae* abscess is becoming a global problem. In accordance with the clinical definition of *Klebsiella pneumoniae* liver abscess syndrome, [1] we propose that *Klebsiella pneumoniae* renal abscess syndrome could be clinically defined as renal abscess secondary to *Klebsiella pneumoniae* infection with metastatic extrarenal involvement.

## 4. Conclusion

Our case emphasizes the importance of considering *Klebsiella pneumoniae* renal abscess syndrome as a part of differential diagnosis in patients presenting with multiorgan septic involvement. Awareness about this new invasive syndrome amongst physicians is important for early diagnosis and management of the infection.
